# More Evidence That Ensemble Music Training Influences Children’s Neurobehavioral Correlates of Auditory Executive Attention

**DOI:** 10.3390/brainsci13050783

**Published:** 2023-05-11

**Authors:** Kylie Schibli, Taylor Hirsch, Gabriel Byczynski, Amedeo D’Angiulli

**Affiliations:** 1 Neuroscience of Imagination Cognition and Emotion Research (NICER) Lab, Carleton University, Ottawa, ON K1S 5B6, Canada; schiblikylie@gmail.com (K.S.); taylorhirsch@cmail.carleton.ca (T.H.); byczynsg@tcd.ie (G.B.); 2 Department of Neuroscience, Carleton University, Ottawa, ON K1S 5B6, Canada; 3 Trinity College Institute of Neuroscience, School of Psychology, Trinity College Dublin, The University of Dublin, D04 V1W8 Dublin, Ireland

**Keywords:** music training, auditory executive attention, sound perception, self-regulation, event-related potentials, auditory Go/NoGo, neuroplasticity, intervention for socioeconomic disadvantaged children

## Abstract

We assessed the neurocognitive correlates of auditory executive attention in low socioeconomic status 9–12-year-old children—with and without training in a social music program (OrKidstra). Event-related potentials (ERPs) were recorded during an auditory Go/NoGo task utilizing 1100 Hz and 2000 Hz pure tones. We examined Go trials, which required attention, tone discrimination and executive response control. We measured Reaction Times (RTs), accuracy and amplitude of relevant ERP signatures: N100-N200 complex, P300, and Late Potentials (LP). Children also completed a screening test for auditory sensory sensitivity and the Peabody Picture Vocabulary Test (PPVT-IV) to assess verbal comprehension. OrKidstra children had faster RTs and larger ERP amplitudes to the Go tone. Specifically, compared to their comparison counterparts, they showed more negative-going polarities bilaterally for N1-N2 and LP signatures across the scalp and larger P300s in parietal and right temporal electrodes; some enhancements were lateralized (i.e., left frontal, and right central and parietal electrodes). Because auditory screening yielded no between-group differences, results suggest that music training did not enhance sensory processing but perceptual and attentional skills, possibly shifting from top-down to more bottom-up processes. Findings have implications for socially based music training interventions in school, specifically for socioeconomically disadvantaged children.

## 1. Introduction

Self-regulation skills refer to the ability to attend to and monitor one’s emotions and maintain emotional homeostasis, remain motivated and attentive, solve problems effectively, pursue short-term or long-term goals, and follow social conventions [1]. It has been proposed that the income-achievement gap can partially be explained by self-regulation, whereby children from higher-socioeconomic status (SES) backgrounds have more opportunities to practice these skills [2]. Children who grow up in poverty often face a variety of stressors, which are known to impede learning in the school setting. For example, chaotic living environments do not allow children to practice self-regulation as they are constantly “on guard”. Therefore, interventions promoting these skills in a stress-free environment may lead to more adaptive coping and better life outcomes. Research has indicated that young children receiving music instruction perform significantly better on self-regulation tasks [3,4]; however, this is untested in a diverse sample.

The current project report presents the preliminary results and implications of a more extensive prospective cohort study conducted over multiple years. Specifically, here we report a comparison of self-regulation ability linked with auditory executive attention in children 9–12 years old participating in a music program, OrKidstra with The Leading Note Foundation [5], with children of similar age and low socioeconomic status. Self-regulation skills are expected to improve as children must cooperate with their peers, take turns, pay attention to the instructor, and follow social norms as they learn to sing and dance in a group. Inhibition control and selective attention were tested using a computer program implementing an auditory task under the Go/NoGo paradigm. Children were invited to respond to one tone at a specific frequency (go-trial) while ignoring the second at a different frequency (no-go trial). This task targets executive attention, working memory, and inhibition, making it a good measure of cognitive self-regulation. In addition, the auditory component of the paradigm requires the participant to perform an element of pitch assessment, positing a perceptual aspect as well. Event-related potentials (ERPs, neural correlates of task performance measured using Task referenced EEG recording) were recorded as they completed the task, along with reaction times and accuracy. In this report, we focused on Go trials, expecting increases in brain activity related to more efficient task perceptual performance based on findings from our previous research. The comparison between Go and NoGo is the basis of another forthcoming report.

In addition, children were asked to complete the Peabody Picture Vocabulary Test-IV (PPVT-IV) to determine if music influences verbal intelligence as demonstrated in previous research [4] and to control for IQ across groups [6,7]. Finally, parents completed the Strengths and Difficulties Questionnaire (SDQ) to assess the child’s wellbeing.

We expected that for some aspects of information processing linked to attention and cognitive self-control (namely, “executive functions”), children enrolled in the OrKidstra music program would perform differently than children not enrolled on the auditory Go/NoGo task (comparison group). We hypothesized that children involved with OrKidstra would show differences in ERPs that are associated with different stages of executive attention processing, such as:

(1) Perceptualattentional processing: ERPs were expected to support behavioural outcomes with children with OrKidstra demonstrating greater amplitudes on the early processing waveforms N1 and N2. A previous study with samples from this population [8] has shown some evidence compatible with this prediction based on spectral analysis (i.e., EEG Power) but no direct evidence in the time domain (i.e., ERPs). Although most of the studies have focused on these early waveforms in the No-Go trials concerning the hypothesis that N1-N2 reflect aspects of response inhibition or conflict monitoring [9,10,11,12,13], there is also a small behavioral and ERP literature which focused on the role of these signatures in the Go trials. The latter studies have shown evidence which suggests that N2, in particular, is related to executive attention relative to response activation and initiation [14,15,16]. Research in typically and atypically developing children has shown evidence that these early components tend to be Frontal and lateralized. Additionally, early ERP features such as the N2 have been implicated in pitch perception, and therefore altered N2 components may represent a change in perceptual processes occurring during stimulus evaluation. [17] Group differences in N2 features may therefore reflect perceptual processing differences. Furthermore, lateralization has been implicated in perceptual differences in processing, for example, increased left-hemispheric activity. Ref. [18] We will therefore consider the possibility that asymmetry differences may represent perceptual differences.
(2) Working memory: Children in the OrKidstra group were expected to show more significant differences for Go trials during stimulus evaluation and categorization on the P3. In particular, one of the key findings in the literature is that music training enhances the rapid plasticity of P3 event-related brain potentials related to the selection of information in working memory via attention [9]. This component is generally found in temporoparietal electrodes [19,20,21].
(3) Executive control related to emitting or inhibiting a response: Children involved with OrKidstra were expected to show greater differences for Go trials on late potentials (Lps): Lp 400–1000 ms, suggesting more regulatory control of top-down processes. This expectation is largely based on the link between self-regulation and linguistic development and the overlap between networks associated with language and music [22]. These networks are generally reflected in activity at mid-frontocentral electrodes [23,24].

We hypothesized that the predicted differences in one, all, or a combination of the above executive functions would partly reflect the effects of practice acquired during musical training. The particular outcome found would clarify whether the changes associated with the music training are general or domain-specific. That is, the influence of the training can be pinpointed to a specific subset of perceptual or cognitive skills and does not transfer across the entire neurocognitive system.

## 2. Methods

### 2.1. Participants

This study involved twenty-six children between the ages of 9–12 years, which included thirteen children (matched age of 10.5 years, seven females) in each of two groups, one with music training (OrKidstra) and a comparison group with limited music training. Data for the entire sample was available for the behavioral measures only. However, usable EEG data was available for twenty of these children (see details on data exclusion below), with eleven children in OrKidstra (mean age 10.8 years; 5 females) and nine in the comparison group (mean age 9.7 years, five females). In the sample of 26 children, 33.3% of the children in the comparison group had some form of musical training outside of OrKidstra. The OrKidstra children had orchestral lessons and intensive practice after school sessions for three hours three times per week. In the subgroup comparison, children who had musical exposure had a range of exposure between one and up to a maximum of two hours per week of lessons and practice sessions. In addition, both groups had similar basic music instructions as part of their school curriculum. This difference was not statistically significant despite a higher percentage of children with parents as musicians in the OrKidstra group (58.3% vs. 25.0%).

We could not establish a reliable, standardized measure of passive exposure to music listening in the respective homes of the two groups. Still, informal discussions with parents did not reveal an impression of a noticeable difference in the frequency or intensity of music listening except in the type of exposure, in that OrKidstra parents seemed more deliberate in the choice and consumption of music played as it seemed to be more selected (i.e., classical, or ethnic music) and seemed to have less occasional radio listening. In the reduced sample (*n* = 20), there was a significant difference between groups for age (t(19) = 2.40, *p* < 0.05), which was used as a covariate for further analyses to “control” for this confound. The two groups were matched for low family socioeconomic status (Level V on the Hollingshead [25] scale) and came from families in the inner-city Ottawa metropolitan area. Most children in both groups were bilingual or multilingual (i.e., English and/or French and/or another language spoken at home). Only one or two children in the OrKidstra and Comparison groups were monolingual. Finally, no handedness differences were revealed by the administration of the Edinburgh Handedness Inventory (EHI) [26]. The EHI is a 20-item measurement scale widely used to screen the self-rated preference or dominance of a person’s right or left-hand use while carrying out everyday tasks. The items comprise tasks of writing, drawing, throwing, using scissors, and a toothbrush, cutting with a knife, using a spoon, using the upper hand when using a broom, striking a match, and opening the lid of a box.

Given that, aside from age distribution, there were no significant demographic, family structure, or screening differences between the sample of 26 children versus the reduced sample of 20 children, we retained the use of all behavioral data. We assumed that the results based on the latter data also applied to the reduced sample.

Children’s participation was conditional on the children’s assent and signed parental consent. This study followed the guidelines outlined by the Canadian Tri-Council policy for ethical research on human subjects [27]. In addition, the research protocol was approved by the institutional, behavioural ethics research board of Carleton University.

### 2.2. Auditory Go/NoGo Task

#### 2.2.1. Pre-Experimental Auditory Tone Detection Screening

A hearing test was performed to assess the normal hearing of the participants using a GS1 61 audiometer (Grason-Stadler, Eden Prairie, MN, USA). This ensured tones could be heard between −10 dB and 25 dB. At a 20 dB Sound Pressure Level, the tones were presented between 500 and 4000 Hz in each left and right ear. All participants were found to have hearing in the normal range.

#### 2.2.2. Experimental Paradigm

**Figure 1:**
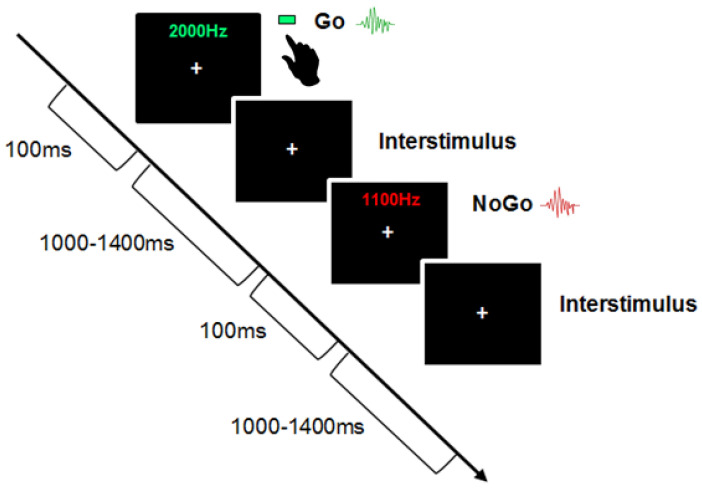
Visual display of the Auditory Go/NoGo task. In the particular example represented in this figure, the Go trial is 2000 Hz, NoGo trial is 1100 Hz. Children were asked to respond to a pure tone at a specific frequency (go-trial) and to withhold their response to the tone played at a different frequency (No-Go trial). The stimulus included two tones: 1100 Hz and 2000 Hz. Each sound was played at the same duration (100 ms) with an interstimulus interval between 1000–1400 ms. The attended sound was presented 70% of the time (Go trial), whereas the unattended sound was presented 30% (No-Go trial). Each block contained 100 trials making a total of 400 trials. Children received a practice session of four blocks of 10 trials (40 trials).

Children were asked to participate in an auditory selective attention task under the Go/NoGo paradigm (Figure 1), where they were asked to respond to a pure tone at a specific frequency (Go-trial) by pressing a button and to withhold their response to a tone played at a different frequency (No-Go trial). The stimulus included two pure tones: 1100 Hz and 2000 Hz. Each sound was played at 100 milliseconds (ms) with an interstimulus interval between 1000–1400 ms. The attended sound was presented 70% of the time (go trial), whereas the unattended sound was presented 30% (no-go trial). Children completed four blocks of 100 trials, making 400 trials. The Go and No-Go sound classification was randomized across blocks, and children were presented with each sound before testing. They were allowed to have the sounds repeated as often as they liked before testing for each block. Children received a practice session of four blocks of 10 trials (total of 40 trials), with the first block providing visual feedback with the word “GO!” flashing on the screen during go trials. The remaining practice blocks and the testing blocks did not involve any feedback. Children were told to keep as still as possible and to look straight ahead at a white cross fixed on a black screen. They were told to press the green button on the response pad with their dominant hand in response to the go sound. Accuracy and reaction time were recorded for the go-trials, and errors on the no-go trials.

### 2.3. Peabody Picture Vocabulary Test–IV

**Figure 2:**
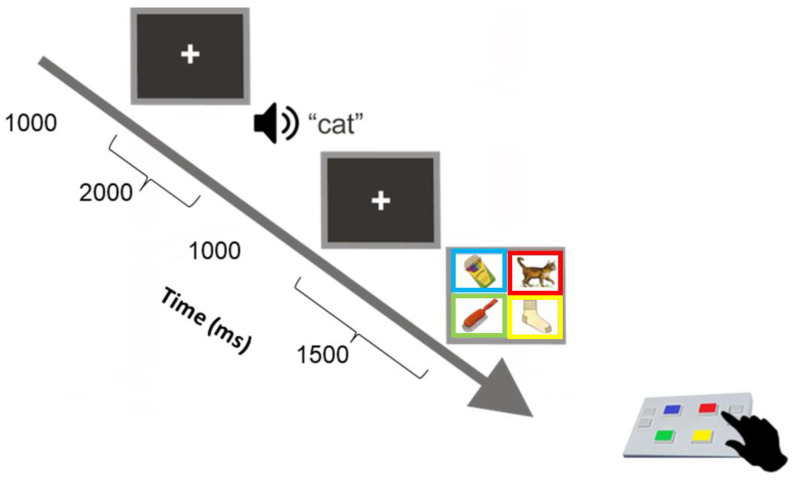
Computerized version of the PPVT-IV [28], which children completed before being capped.

A computerized version of the standard Picture Peabody Vocabulary Test (PPVT-VI) was used to measure the child’s receptive vocabulary and word comprehension [28]. The PPVT is an accepted measure of word comprehension and semantic elaboration. The strong relationship between the processes measured by the PPVT-IV and language comprehension [29,30] show correlations between the PPVT-IV and kindergarten language comprehension are very strong (median r > 0.65, see [14]). Therefore, performance on the PPVT-IV would likely reflect the child’s preschool level of linguistic processing. This has been confirmed by studies in aphasiology [31,32,33], intelligence [34], and clinical neuropsychology [34] in children and adults. The PPVT-IV includes 19 sets, with 24 target words per set presented aurally and a corresponding four-colour picture display (see Figure 2). (The task consists of practice trials where participants are asked to identify two concrete words by correctly selecting the target picture from four pictures. The practice items are intended to teach the child how to use the response pad to make a correct response. The experiment trials begin if the child responds correctly to two training items. If the child responds incorrectly to either of the first two training items, a reduced level of training is first administered. All children responded correctly to the first set of training items).

As represented in Figure 2, each picture of the display set showed a rectangular frame with the colour corresponding to a button colour at the same spatial location on the response pad as the one shown on the screen. Children were instructed to listen to a word presented at 60 dBHL over insert earphones and then select the picture that best illustrated the meaning of the heard (target) word by pressing the corresponding button on the response pad. Each colour-coded button on the pad had an equal probability of response (25% of 24 words per block). The pre-recorded words were of an English-speaking female voice with an average fundamental frequency of 250 Hz. Each new trial was self-initiated by pressing any button on the response pad. A set was completed successfully until three consecutive words were responded to incorrectly; the task was programmed to discontinue when this occurred, and that final set was considered the maximum performance level assigned to the participant.

The presentation sequence of the stimulus sets was arranged in order of decreasing concreteness and increasing complexity/abstractness (i.e., norm-based critical range going from concrete to more abstract and complex) so that the task could be calibrated to, and assess, the child’s vocabulary level as reflected by the norm-based standardized critical range (standard scores). All children performed at or above age-appropriate levels, and no systematic differences were detected between the music and the comparison group.

### 2.4. The Strengths and Difficulties Questionnaire (SDQ)

The SDQ is a brief behavioural screening questionnaire used with children ranging from 3–17 years of age [35]. The questionnaire is used to assess children’s mental health and can be completed by children and young people themselves, by their parents or by their teachers. Considering there were children younger than 11 in our study, wording from the parent version for children 4–10 years was used. The children’s guardians/parents were asked to complete the questionnaire electronically. The parent version 10–17 years is composed of the same questions and categories with only minor changes in terminology (for example, “children” is replaced by “youth”). Given our sample was on the lower end of the age range (9–12 years), we did not feel it necessary to provide both options. The parent and teacher-rated SDQ consists of 25 items rated on a 3-point Likert scale (not true, somewhat true, and certainly true), with a mixture of positive and negatively phrased items. The 25 items are designed to be divided between the following five sub-scales. In our study, parents or guardians were asked to respond to questions assessing their child’s psychological attributes on:

1. Emotional symptoms (5 items; for example: “Often unhappy, depressed or tearful”).
2. Conduct problems (5 items; for example: “Steals from home, school or elsewhere”).
3. Hyperactivity/inattention (5 items; for example: “Restless, overactive, cannot stay still for long”).
4. Peer relationship problems (5 items; for example: “Often fights with other children or bullies them”).
5. Prosocial behaviour (5 items; for example: “Considerate of other people’s feelings”).

Responses on scales 1–4 are added to obtain a total difficulties score. A follow-up questionnaire further screened whether the child’s difficulties have an impact emotionally, socially, behaviourally and with concentration.

### 2.5. Event-Related Potentials (ERPs)

ERPs refer to a change in brain activity following an internal or external sensory stimulus. This is a non-invasive technique with an effective temporal resolution providing an accurate measurement of when processing takes place in the brain. ERPs are characterized by their latency following stimulus presentation in milliseconds, polarity (negative or positive), and spatial topography. A common issue in ERP studies is whether the observed data have sufficient trials to support statistical analysis. The background noise in any ERP for any individual can vary. To see the brain’s response to a stimulus, the experimenter must conduct many trials and average the results together, causing random brain activity to be averaged out and the relevant waveform to remain. Consequently, these signals are calculated by averaging the summed potentials following the repetition of stimulus presentation during a task. They are often used in research on human cognition [36].

When overviewing the (large) Go/NoGo literature, as specifically applied to our hypotheses, the N1 and P2 are components commonly present during the early sensory processing of stimuli during the auditory Go/NoGo task. The N1 was found to have a frontocentral maximum maximal in the right hemisphere among 10-year-old children and was larger for NoGo stimuli. In contrast, the P2 was larger to Go than NoGo stimuli and had a parietal-central maximum [37]. Developmental research involving a Go/NoGo task generally centers on the N1-2 [38,39] and P3 [40,41]. It has been suggested that the N1 and N2 in children can be associated with attention and sensory-perceptual processing [42], whereas the P3 is commonly associated with stimuli identification and working memory [43]. ERPs in the later stage of processing are thought to reflect a planned response to a desired goal, and changes related to them can reflect aspects of withholding or emitting a valid response. Larger differences between peaks on Go and NoGo trials indicate early attention (N1/N2), working memory (P3), and planned responses (N4/P6). Our ERP analysis focused specifically on the time windows of these signatures.

#### EEG/ERP Data Acquisition and Recording

Participants were fitted with a 32-channel Brain Vision actiCAP electroencephalography (EEG) cap (actiCAP, Brain Vision LLC, Morrisville, NC, USA). All the inserted electrodes in the cap conformed to a referential montage and the International 10–20 EEG electrode placement system [22]. In addition, two additional drop-down electrodes were placed on the outer canthus of the eyes, along with the ground electrode, which was placed on the participant’s left collarbone. Given the objectives of the present work, we excluded orbifrontal and occipital electrodes for the analysis.

Electrophysiological signals were amplified (gain of 10; Range of ±200 μV, or 400 μV peak-to-peak; Accuracy 29.80 nV/LSB) and low pass filtered at 500 Hz via a Neuroscan SynAmps RT (Compumedics, El Paso, TX, USA) with a sampling rate of 1000 Hz. Acquisition filters were single-pole Butterworth, 6 dB per octave, 3 dB down at 500 Hz. All electrodes were referenced to a separate reference electrode, and all data were re-referenced to a common average reference.

Resting EEGs (i.e., pre- and post-experiment 2-min open/closed eyes) were clinically unremarkable in all children. ERPs were recorded from the participants during the entire duration of the Go/No-Go task. For pre-processing, ERPs were baseline corrected relative to prestimulus interval and averaged separately for each stimulus type and condition for each electrode with an epoch of −200 ms prestimulus to 1000 ms post-stimulus. Trials contaminated by excessive peak-to-peak deflection (i.e., >100 μV or <−100 μV) at non-ocular electrode sites were excluded from the average. The proportion of rejected trials was less than 10% after artifact correction and removal. The post-data acquisition average signal from the electrodes was amplified and digitized with filter settings at 0.15 Hz (high pass) and 100 Hz (low pass). Horizontal eye movements were monitored with electrodes from a split bipolar electrode positioned at the outer canthi at HEOG. All impedances were kept under 5 kΩ. The electrode locations were mapped and analyzed using Brain Electric Source Analysis (BESA V6) [44].

Data from two children from the OrKidstra group and four from the Comparison group (6 children) were discarded because they were incomplete and/or presented very noisy background EEGs.

Eye-movement calibration was completed before Go/No-Go testing. Ocular correction was then performed using Principal Component Analysis and the BESA Surrogate Model (BR_Brain Regions_LR.bsa). Finally, artifact correction for blinks, horizontal and vertical eye movements, and interpolating noisy EEG raw data for specific electrodes were performed based on the 32-channel configuration (fit threshold of artifacts classification to BESA model was R2 ≥ 0.80).

## 3. Results

### 3.1. Behavioral Findings

An initial analysis of the auditory tone detection screening scores showed no difference in tone discrimination between the two groups (median z = 0.41, median *p* = 0.69).

Subsequently, to control for age differences and enhance statistical power, we sampled-matched the children according to sex and age (age difference within one year ± 6 months), thereby obtaining thirteen sample-matched pairs. Subsequently, we submitted the means of the behavioral data to a wholly nested 2 × 2 ANOVA having as within-subjects (repeated measures) factors Tone frequency (1100 Hz versus 2000 Hz) and Group (OrKidstra versus Comparison); the within-subjects (repeated measures) effects were adjusted using Greenhouse-Geisser correction.

**Figure 3:**
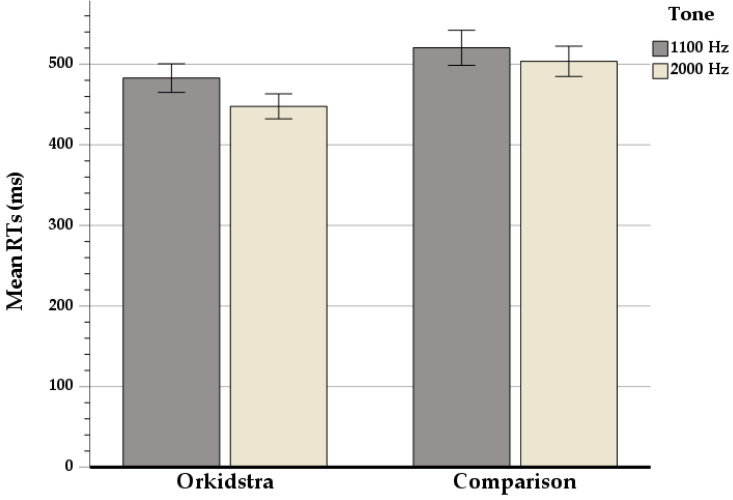
Difference of mean reaction times between OrKidstra and Comparison groups on Go trials for the two conditions: 1100 Hz and 2000 Hz. Error bars represent ±1 standard error.

For the reaction time (RT) data, which is shown in Figure 3, the ANOVA analysis revealed a main effect of Tone frequency associated with the Go trials in that RTs were faster for 2000 Hz than for 1100 Hz tones (F(1,12) = 7.01; *p* < 0.05), but no interaction effect. Importantly, the other main effect of the Group also showed a significant, strong effect (F(1,12) = 5.11; *p* < 0.05), indicating that it seems quite safe to infer that, in all likelihood, the Orkidstra children responded relatively faster than their counterparts.

Accuracy, correct rejections, missed responses, and false alarm rates were not significantly different between the two groups. In addition, the interaction between Group (OrKidstra versus Comparison) and Tone condition (1100 Hz versus 2000 Hz) was insignificant.

No significant differences existed between groups for performance on the PPVT-IV or parental responses on the SDQ. In addition, we found no reliable sex differences. Finally, we verified that there were no differences in patterns of results between the complete behavioral dataset (*n* = 26) compared to the analysis based on the reduced dataset (*n* = 20). Although the result showed no differences in the patterns of results, they indicated an increase in statistical power by including the behavioral data from all 26 children.

### 3.2. Neuro-Electrophysiological Findings

We utilized an analytical strategy that focused specifically on the accuracy and reliability of the mean ERP signals at electrode sites matched between the two groups. Thus, the unit of analysis or the “subjects” in the database were the electrodes. This within-subject approach is usually traditionally known as a by-item or item-wise analysis [45,46] or, in keeping with EEG terminology, an electrode-wise analysis [47]. The item-wise approach corresponds to a random-effects model on the items and a fixed-effects model on subjects. Therefore, the effects of statistical tests can be generalized to new things and tasks from the same subjects but cannot be used to make a reliable prediction for new subjects that would be generalized to the population [48]. The mean-wise group approach is ordinarily used to obtain the highest possible effect. The item-wise approach, conversely, centers around the pursuit of replicable effects based on weak but stable interindividual correlation coefficients, that is, on relatively homogeneous intraindividual variance. The weak correlation for an item is generally due to excessive interindividual noise. To reduce such noise heterogeneity, in our study, the participants’ data were first partitioned according to time-series bins with the same data density per interval of time (25 milliseconds) by averaging the participants’ quantile rank (as in [49]). Successively, bin-by-bin means were estimated for each electrode (i.e., across participants). This electrode-wise approach results in a wholly nested within-subject design that tends to homogenize (i.e., limit heterogeneity of) the combined inter- and intraindividual variance.

**Figure 4:**
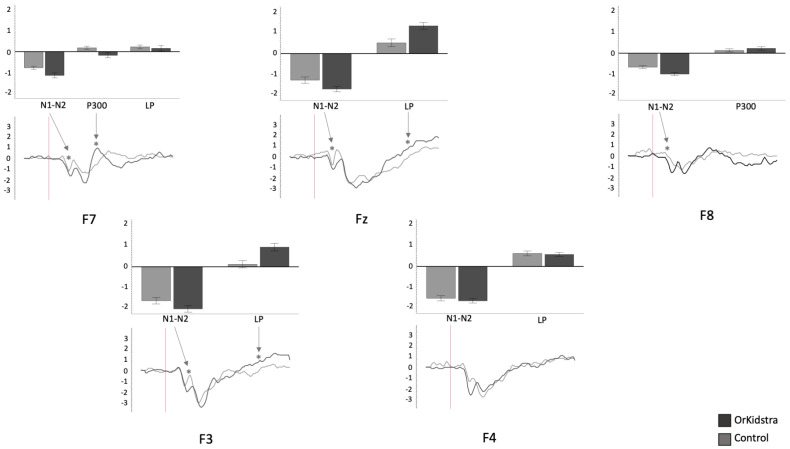
Graphs of frontal electrode ANOVAs (F7, F3, Fz, F4, F8) showing the averages of estimated marginal means across participants, divided by group types (line graphs) as well as the polarity averages across participants divided by ERP component type (bar graphs). Asterisks (*) mark moments where the averaged data for groups was significantly different from one another. For each graph, polarity is measured in millivolts (mV) and time in milliseconds (ms). Bars represent standard errors.

**Figure 5:**
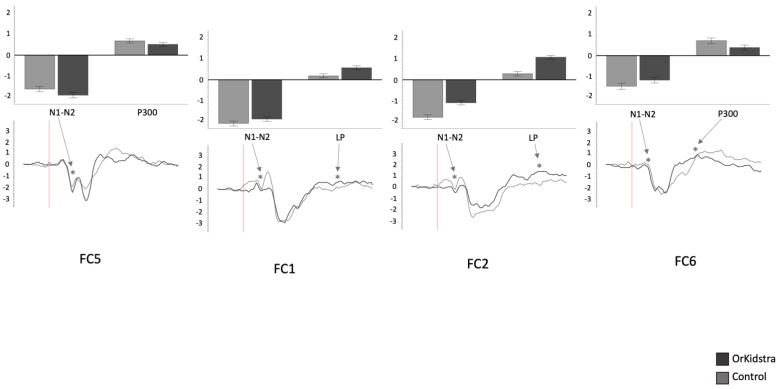
Graphs of frontocentral electrode ANOVAs (FC5, FC1, FC2, FC6) showing the averages of estimated marginal means across participants, divided by group types (line graphs) as well as the polarity averages across participants divided by ERP component type (bar graphs). Asterisks (*) mark moments where the averaged data for groups was significantly different from one another. For each graph, polarity is measured in millivolts (mV) and time in milliseconds (ms). Bars represent standard errors.

**Figure 6:**
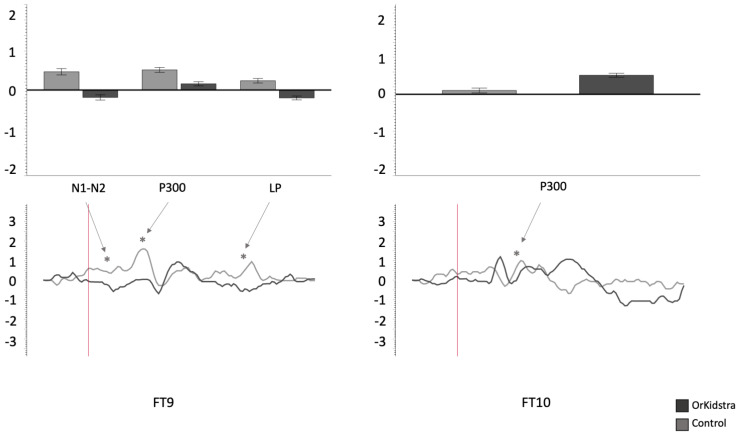
Graphs of frontotemporal electrode ANOVAs (FT9, FT10) showing the averages of estimated marginal means across participants, divided by group types (line graphs), as well as the polarity averages across participants divided by ERP component type (bar graphs). Asterisks (*) mark moments where the averaged data for groups was significantly different from one another. For each graph, polarity is measured in millivolts (mV) and time in milliseconds (ms). Bars represent standard errors.

**Figure 7:**
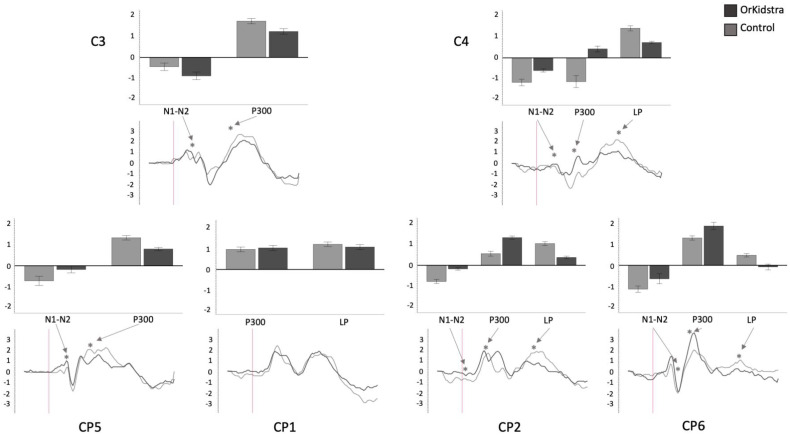
Graphs of central and centro parietal electrode ANOVAs (CP5, C3, CP1, CP2, C4, CP6) showing the averages of estimated marginal means across participants, divided by group types (line graphs) as well as the polarity averages across participants divided by ERP component type (bar graphs). Asterisks (*) mark moments where the averaged data for groups was significantly different from one another. For each graph, polarity is measured in millivolts (mV) and time in milliseconds (ms). Bars represent standard errors.

**Figure 8:**
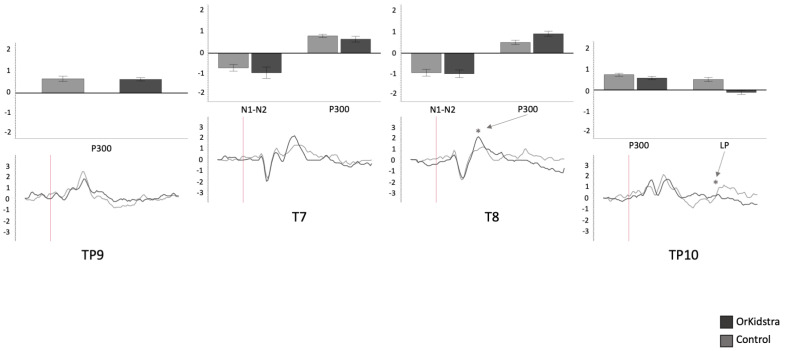
Graphs of temporal and temporoparietal electrode ANOVAs (TP9, T7, T8 TP10) showing the averages of estimated marginal means across participants, divided by group types (line graphs) as well as the polarity averages across participants divided by ERP component type (bar graphs). Asterisks (*) mark moments where the averaged data for groups was significantly different from one another. For each graph, polarity is measured in millivolts (mV) and time in milliseconds (ms). Bars represent standard errors.

**Figure 9:**
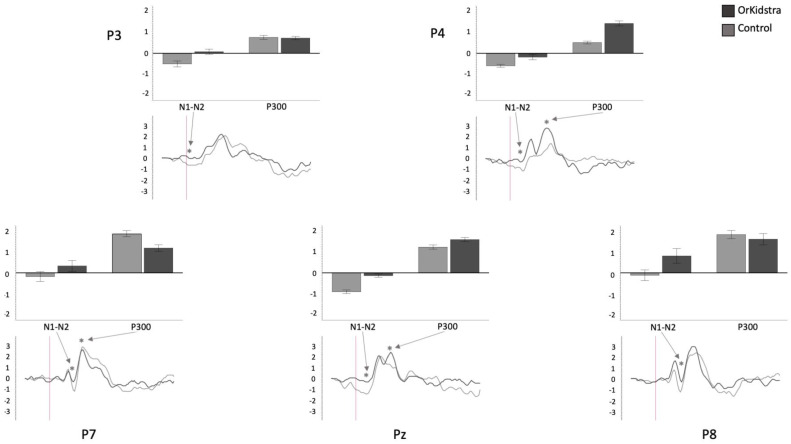
Graphs of parietal electrode ANOVAs (P7, P3, Pz, P4, P8) showing the averages of estimated marginal means across participants, divided by group types (line graphs) as well as the polarity averages across participants divided by ERP component type (bar graphs). Asterisks (*) mark moments where the averaged data for groups was significantly different from one another. For each graph, polarity is measured in millivolts (mV) and time in milliseconds (ms). Bars represent standard errors.

The 25-millisecond binned average microvolt measurements for each electrode and in each group were divided into the time windows which defined our ERP component of interest (N1-N2: 50–230 ms; P300: 240–380 ms; LP: 500–1000 ms). Successively, we ran separate univariate ANOVAs on the binned averages with Group (Orkidstra versus Comparison) X ERP Component (N1-N2 versus P300 versus LP) for each electrode. The results of all these analyses are shown graphically in Figure 4, Figure 5, Figure 6, Figure 7, Figure 8 and Figure 9; Table 1 reports the statistics for the specific ANOVA contrasts that were significant for the main effects of the Component factor or interactions subsuming non-significant main results. The ERP data show several differences between the Orkidstra and the Comparison group. To facilitate the interpretation of these complex findings, we present a summary in Table 2.

Orkidstra children showed more negative going polarities across the scalp than the comparison group except for parietal and right temporal electrodes, which showed larger P300. However, LPs were almost always more positive in comparison to children. Although most of these effects occurred bilaterally, there were sporadic lateralization effects, especially in the left frontal and right central and parietal electrodes (see Table 2).

## 4. Discussion

Our findings demonstrate that children involved with OrKidstra are faster than a comparison group at responding to Go stimuli (at 1100 Hz and 2000 Hz), supported by a greater activation during early potentials, such as the N1 and N2. These findings suggest musical training with OrKidstra influences early sensory processing leading to faster behavioral performance. A plausible and parsimonious interpretation consistent with previous results is that a slower reaction time on the auditory Go/NoGo task may reflect difficulty discriminating effectively between the two stimuli leading to focused attention on the NoGo tone, mediated by perceptual processing speed [50]. Children in OrKidstra had a faster response time to the Go tones and a greater degree of early neuronal processing in response to both frequencies. These effects were apparent within and between groups, suggesting a practice effect leading to an improved ability to discriminate between sounds. It is possible that as children develop more practice in listening and deciphering sounds, they shift from recruiting top-down frontal to more “perceptual” centroparietal processes. As children learn to shift their focus through musical training, attentional processing becomes more automatic and is processed at a lower level in the neural network cognitive system. That is, they seem to use a more perceptual mode of processing.

One possible explanation is that with music training, pitch perception changes from comparing pitches to each other to evaluating the go or no-go response to evaluating pitch elements [8,51]. With this interpretation, our results indicate that music training alters the processing of pitches and, taken together with earlier ERP signatures, might explain the behavioral effects of faster RTs. In addition, research has shown that more significant N2 responses are associated with ‘ratio simplicity’. The cortical activity responding to pitch discrimination represents perceptual qualities such as pleasantness and/or consonance [52]. Here we report that N1-N2 responses in the frontal regions (F3, F7, Fz, F4, F8) showed larger amplitudes in the Orkidstra group compared to the comparison group, positing support for different approaches to pitch assessment between the groups. Ultimately, this agrees with the notion that music training produces altered pitch discrimination methods that favor the perception of pitch quality (i.e., the dissonance between pitches). This altered perceptual processing may result in a more streamlined or efficient evaluation method to inform response, resulting in improved performance.

In agreement with previous work that suggests increased left-asymmetry over T7 reflects recruitment of the supramarginal gyrus (SMG). This region contributes to pitch memory in non-musicians. Our results showed a significantly larger P300 amplitude in the comparison group [53]. This may reflect the alternate mode of perceptual processing in non-musicians, such that non-musicians rely on remembering the ‘Go’ or ‘NoGo’ pitch and comparing it to the presented one. We, therefore, interpret our results as evidence that there is a difference in perceptual pitch processing between the groups, whereby musicians rely on more perceptual qualities. In contrast, the comparison group instead may depend on a comparison approach.

However, an important mechanism that may underlie the reliance on faster perceptual processing in children with musical training could be partly explained by a shift in early auditory selective attention. Our screening testing with the audiometer showed no difference between the sensory processing of the two groups. This is possible because the task was a passive tone detection involving no selection. The shift from late to early processing may free cognitive executive resources for more flexible attention control and allow children to do other tasks (possibly simultaneously) in working memory. Indeed, this evidence again suggests that the differences between the two groups lie in the perceptual processing of pitch evaluation. If this result were confirmed with a larger sample, it would shed some light on the underlying memory processes that allow children to automatize and make more efficient neural responses to musical and sound stimuli with practice. This opens the field to novel intriguing phenomena that await further scientific investigation.

## 5. Limitations and Future Directions

Although restricted to Go trials, our findings suggest a difference in early executive attention between children involved with OrKidstra and a comparison group. This effect is present even though 33% of children in the comparison group had some form of music experience. The latter supports the interpretation favouring a direct effect of the OrKidstra training. However, assuming these findings can be projected to a broader population is premature, given the small sample size. Efforts are ongoing to determine the generalizability of our preliminary results, and to confirm the ERPs differences found in this preliminary analysis. In addition, we plan to use a series of alternative methods (i.e., transcranial direct current stimulation, positron emission tomography) combined with EEG to validate some of the findings further and relate them to metabolic functions associated with brain growth and development.

Despite no significant differences in the SDQ between groups, there was an effect in the expected direction in the area of conduct problems (*p* = 0.137), with children involved with OrKidstra demonstrating fewer difficulties. This is worth examining further and may help us understand the positive social/emotional impact attributed to participation in the OrKidstra program.

## 6. Conclusions

In conclusion, the current study examined the effect of participation in a social music and movement program, OrKidstra, on children’s self-regulating ability. The preliminary findings indicate that children participating in the OrKidstra program seem to benefit from the training in perceptual processing, reflected at both behavioral and neural levels. However, the validity of the present findings needs to be confirmed with a larger sample and other pediatric and age populations, including different forms of music training and instruction.

The present findings, however, do add to our previous work examining other aspects of the effects of this particular music instruction program [8,54]. This study further contributes to the converging evidence that socially-based intensive music training such as OrKidstra can potentially benefit children’s brain and developmental plasticity.

Indeed, despite its limitations, the present study showed evidence suggesting that socially based music training offered to socioeconomically disadvantaged (i.e., low socioeconomic status) children changed the processes associated with sound perception at the neural level. The pattern of results suggested evidence of improved sound perception, with different approaches to task processing between subgroups of children with similar low SES. One such difference may include the change from pitch comparison to more sophisticated and music-rooted evaluation, such as dissonance or pitch quality. The present study contributes evidence suggesting the use of ERPs as a possible assessment tool for developmental and educational interventions in socioeconomically disadvantaged and underserved children. Further, it highlights the validity of the socially based ensemble music training principle as a potential candidate for ameliorative supportive interventions for low SES children. Thus, our findings give additional support, in the form of “hard” brain correlates data, to the well-known positive influences of musical schools in low-income countries and particularly in the most disadvantaged groups—like “El Sistema” in Venezuela [55], and “Sol del Illimani” in the outskirts of Santiago Chile [56]. This suggests that (with some apparent cultural adaptation) the same similar music education principles can apply to other contexts of the globe and positively influence children’s lives.

## Figures and Tables

**Table 1:** Table of Electrode Contrasts.

Effects	F-Contrast (1, 74)	*p*	Partial Eta^2^	Observed Power
F7	11.53	0.001 *	0.135	0.918
F3	10.81	0.002 *	0.205	0.895
Fz	16.34	<0.001 *	0.220	0.978
F4	2.20	0.143	0.034	0.309
Group × F4	0.410	0.524	0.006	0.097
F8	3.97	0.052	0.071	0.498
Group × F8	13.33	<0.001 *	0.204	0.948
FC5	7.12	0.010 *	0.105	0.748
FC1	87.45	<0.001 *	0.570	1.000
FC2	431.4	<0.001 *	0.855	1.000
FC6	0.049	0.825	0.001	0.055
Group × FC6	14.79	<0.001 *	0.172	0.967
FT9	79.87	<0.001 *	0.273	0.986
FT10	18.00	<0.001 *	0.462	1.000
C3	69.98	<0.001 *	0.583	1.000
C4	33.99	<0.001 *	0.382	1.000
CP5	0.001	0.978	0.000	0.050
Group × CP5	43.19	<0.001 *	0.444	1.000
CP1	0.277	0.601	0.006	0.081
Group × CP1	3.461	0.069	0.069	0.445
CP2	17.43	<0.001 *	0.172	0.985
CP6	5.122	0.027	0.082	0.605
Group × CP6	32.86	<0.001 *	0.536	1.000
T7	3.671	0.059	0.079	0.475
Group × T7	0.174	0.679	0.174	0.069
T8	6.797	0.013 *	0.152	0.719
TP9	0.127	0.723	0.004	0.064
TP10	51.77	<0.001 *	0.384	1.000
P7	1.783	0.191	0.054	0.253
Group × T7	79.10	<0.001 *	0.718	1.000
P3	17.30	<0.001 *	0.198	0.984
Pz	80.69	<0.001 *	0.627	1.000
P4	71.16	<0.001 *	0.551	1.000
P8	7.439	0.012 *	0.227	0.741

Note: * Threshold significance is *p* < 0.023 after mean False Discovery Rate (FDR) correction.

**Table 2:** Summary Table of Differences ERP Components By Electrode.

Electrode Homologues		ERP Components	
F7	F8	N1-N2, P300	N1-N2, P300
F3	F4	N1-N2, LP	/
FC5	FC6	N1-N2	N1-N2, P300 *
FC1	FC2	N1-N2, LP	N1-N2, LP
FT9	FT10	N1-N2 *, P300, LP *	P300
C3	C4	N1-N2, P300	N1-N2, P300, LP *
CP5	CP6	N1-N2, P300	N1-N2, P300, LP *
CP1	CP2	/	N1-N2 *, P300 *, LP *
T7	T8	/	P300 *
TP9	TP10	/	LP *
P3	P4	N1-N2	N1-N2, P300 *
P7	P8	N1-N2, P300 *	N1-N2

Note: Only results from ERP components that were significantly different between participant groups (Orkidstra & Comparison) are shown in the table. Components that resulted from one electrode but did not correspond to its homologous electrode are given an asterisk (*).

## Data Availability

Data are available upon request from AD’A, provided the purpose of the request is used in accordance with international intellectual property and copyright regulations.
